# Supplementary material for: The cytoskeletal motor proteins Dynein and MyoV direct apical transport of Crumbs
Source: Dev Biol. 2020 Mar 15;459(2):126–37. doi: 10.1016/j.ydbio.2019.12.009 (PMC7090908; doi:10.1016/j.ydbio.2019.12.009)
Supplement: Multimedia component 1 [file mmc1.docx]

**Supplementary Figure Legends**

**Figure S1. Disruption of apical microtubule and F-actin transport affects Crb but does not strongly affect the polarization of aPKC in the *Drosophila* follicle cell epithelium.**

1. Egg chambers (st 7/8) immunostained for Dlg and aPKC show normal apical localization of aPKC even after expression of *tj.Gal4 UAS.dynein-RNAi.* In contrast to aPKC, Crb-GFP is lost from the apical domain upon expression of *tj.Gal4 UAS.dynein-RNAi* in double labelling experiments (three examples shown)*.* Scale bars approximately 10µm.
2. Egg chambers (st 7/8) immunostained for aPKC, Dlg or Crb showing normal localization of aPKC despite trapping of Crb in apical endosomes upon expression of *tj.Gal4 UAS.MyoV-GT-GFP*, and failure to maintain apical localization of those same endosomes upon treatment with the microtubule depolymerizing drug Colchicine. Scale bars approximately 10µm.
3. Control Crb-GFP apical localization for comparison with (A). See also Figs 1&2.

**Figure S2. Apical Spectrin acts in parallel with the Exocyst to promote apical delivery of Crb**

Induction of MARCM clones (GFP+) mutant for *sec15[1]* and expressing *UAS.karst-RNAi* result in massive accumulation of endosomal Crb in a stage 11 egg chamber. The genotypes shown in these experiments are: *w hs.flp UAS.GFPnls tub.Gal4/+; UAS.karst-RNAi/+; FRT82B sec15^1^ / FRT82B tub.Gal80* and *w hs.flp UAS.GFPnls tub.Gal4/+; UAS.alpha-spectrin-RNAi/+; FRT82B sec15^1^ / FRT82B tub.Gal80*

Scale bars approximately 10µm.
